# Extent of publication bias in different categories of research cohorts: a meta-analysis of empirical studies

**DOI:** 10.1186/1471-2288-9-79

**Published:** 2009-11-26

**Authors:** Fujian Song, Sheetal Parekh-Bhurke, Lee Hooper, Yoon K Loke, Jon J Ryder, Alex J Sutton, Caroline B Hing, Ian Harvey

**Affiliations:** 1 School of Medicine, Health Policy and Practice, University of East Anglia, Earlham Road, Norwich, NR4 7TJ, UK; 2 School of Allied Health Professions, University of East Anglia, Earlham Road, Norwich, NR4 7TJ, UK; 3 Department of Health Sciences, University of Leicester, University Road, Leicester, LE1 7RH, UK; 4 Watford General Hospital, 60 Vicarage Road, Watford, Hertfordshire, WD18 0HB, UK

## Abstract

**Background:**

The validity of research synthesis is threatened if published studies comprise a biased selection of all studies that have been conducted. We conducted a meta-analysis to ascertain the strength and consistency of the association between study results and formal publication.

**Methods:**

The Cochrane Methodology Register Database, MEDLINE and other electronic bibliographic databases were searched (to May 2009) to identify empirical studies that tracked a cohort of studies and reported the odds of formal publication by study results. Reference lists of retrieved articles were also examined for relevant studies. Odds ratios were used to measure the association between formal publication and significant or positive results. Included studies were separated into subgroups according to starting time of follow-up, and results from individual cohort studies within the subgroups were quantitatively pooled.

**Results:**

We identified 12 cohort studies that followed up research from inception, four that included trials submitted to a regulatory authority, 28 that assessed the fate of studies presented as conference abstracts, and four cohort studies that followed manuscripts submitted to journals. The pooled odds ratio of publication of studies with positive results, compared to those without positive results (publication bias) was 2.78 (95% CI: 2.10 to 3.69) in cohorts that followed from inception, 5.00 (95% CI: 2.01 to 12.45) in trials submitted to regulatory authority, 1.70 (95% CI: 1.44 to 2.02) in abstract cohorts, and 1.06 (95% CI: 0.80 to 1.39) in cohorts of manuscripts.

**Conclusion:**

Dissemination of research findings is likely to be a biased process. Publication bias appears to occur early, mainly before the presentation of findings at conferences or submission of manuscripts to journals.

## Background

Synthesis of published research is increasingly important in providing relevant and valid research evidence to inform clinical and health policy decision making. However, the validity of research synthesis based on published literature is threatened if published studies comprise a biased selection of the whole set of all conducted studies [1].

The observation that many studies are never published was termed "the file-drawer problem" by Rosenthal in 1979 [2]. The importance of this problem depends on whether or not the published studies are representative of all studies that have been conducted. If the published studies are a random sample of all studies that have been conducted, there will be no bias and the average estimate based on the published studies will be similar to that based on all studies. If the published studies comprise a biased sample of all studies that have been conducted, the results of a literature review will be misleading [3]. For example, the efficacy of a treatment will be exaggerated if studies with positive results are more likely to be published than those with negative results.

Publication bias is defined as "the tendency on the parts of investigators, reviewers, and editors to submit or accept manuscripts for publication based on the direction or strength of the study findings" [4]. The existence of publication bias was first suspected by Sterling in 1959, after observing that 97% of studies published in four major psychology journals provided statistically significant results [5]. In 1995, the same author concluded that the practices leading to publication bias had not changed over a period of 30 years [6].

Evidence of publication bias can be classified as direct or indirect [7]. Direct evidence includes the acknowledgement of bias by those involved in the publication process (investigators, referees or editors), comparison of the results of published and unpublished studies, and the follow-up of cohorts of registered studies [8]. Indirect evidence includes the observation of disproportionately high percentage of positive findings in the published literature, and a larger effect size in small studies as compared with large studies. This evidence is indirect because factors other than publication bias may also lead to the observed disparities.

In a Health Technology Assessment (HTA) report published in 2000, we presented a comprehensive review of studies that provided empirical evidence of publication and related biases [8]. The review found that studies with significant or favourable results were more likely to be published, or were likely to be published earlier, than those with non-significant or unimportant results. There was limited and indirect evidence indicating the possibility of full publication bias, outcome reporting bias, duplicate publication bias, and language bias. Considering that the spectrum of the accessibility of research results (dissemination profile) ranges from completely inaccessible to easily accessible, it was suggested that a single term 'dissemination bias' could be used to denote all types of publication and related biases [8].

Since then, many new empirical studies on publication and related biases have been published. For example, Egger et al provided further empirical evidence on publication bias, language bias, grey literature bias, and MEDLINE index bias [9], and Moher et al evaluated language bias in meta-analyses of randomised controlled trials [10]. Recently, more convincing evidence on outcome reporting bias has been published [11-13]. In addition, a large number of studies of publication bias in conference abstracts have been published [14]. As this new empirical evidence may strengthen or contradict the empirical evidence included in the previous review, we have updated our review of dissemination bias, by synthesizing findings from newly and previously identified studies [15]. This paper focuses on findings from a review of cohort studies that provided direct evidence on dissemination bias by investigating the association between publication and study results.

## Methods

### Criteria for inclusion

**Figure 1:**
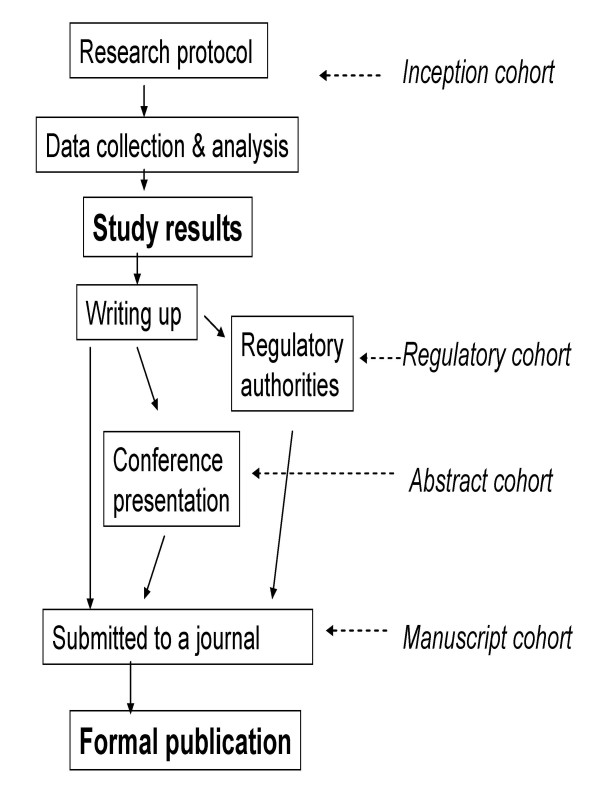
**Pathway from research protocol to journal publication and categories of research cohorts**.

This review included any studies that tracked a cohort of studies and reported the rate of publication by study results. The relevant empirical studies may have tracked a cohort of protocols approved by research ethics committees, registered by research funding bodies, submitted to regulatory authorities, presented at conferences, or submitted to journals. Primary studies included in such cohorts could be clinical trials, observational or basic research. We separated the cohorts into four subgroups according to stages in a simplified pathway of research publication from inception to the journal publication (Figure 1). A study that followed up a cohort of research from the beginning (even if retrospectively) was termed an inception cohort study. A regulatory cohort study refers to a study that examined formal publication of clinical trials submitted to regulatory authorities (such as the US Food and Drug Administration or similar). An abstract cohort study investigated the subsequent full publication of abstracts presented at conferences. A manuscript cohort study followed the publication fate of manuscripts submitted to journals. Studies that did not provide sufficient data to compare the rates of publication between studies with different results were excluded.

### Literature Search strategy

The identification of cohort studies for this review was conducted as part of a comprehensive search for empirical and methodological studies on research dissemination bias. We searched MEDLINE, the Cochrane Methodology Register Database (CMRD), EMBASE, AMED and CINAHL to August 2008 (see Additional file 1). PubMed, PsycINFO and OpenSIGLE were searched in May 2009 to locate more recently published studies. References (titles with or without abstracts) identified by the MEDLINE and CMRD searches were examined independently by two reviewers, while those from other databases were assessed by one reviewer. The reference lists of retrieved studies and reviews were examined to identify additional studies.

### Data Extraction and Synthesis

Data extraction included type of cohort, clinical speciality, design of studies, duration of follow-up, definition of study results, and the rate of publication by study results. One reviewer extracted data directly into tables, which were checked by a second reviewer.

The outcome, study publication, was usually defined as full publication in a journal, but study results were often categorised differently between included cohorts. We used the classifications 'statistically significant (p ≤ 0.05)' versus 'non-significant (p > 0.05)' or 'positive' versus 'non-positive'. Positive results included results that were considered to be 'significant', 'positive', 'favourable', 'important', 'striking', 'showed effect', or 'confirmatory', while non-positive results were labelled as being 'negative', 'non-significant', 'less or not important', 'invalidating', 'inconclusive', 'questionable', 'null', or 'neutral'.

The validity of the included cohort studies was not formally assessed in this review, due to a lack of reliable tools for assessment of methodological studies. However, we tried to identify and summarise the main methodological limitations in the included studies.

Data from the included studies were first summarised in narrative form. The odds ratio was used as the outcome statistic to measure the association between study results and subsequent publication. In existing reviews of cohort studies of publication bias, results from different studies have been quantitatively combined [14,16,17], although significant heterogeneity across individual studies has lead to some controversy [18]. We felt it helpful to provide pooled estimates after separating the included cohort studies into appropriate subgroups, in order to improve statistical power and generalisability. Results from individual studies were quantitatively pooled using random-effects meta-analysis. Heterogeneity within each subgroup was measured using the *I*^2 ^statistic, considering heterogeneity to be moderate or high when *I*^2 ^is greater than 50% [19]. Rucker et al pointed out that, given the same between-study variance, the value of *I*^2 ^will increase rapidly as the sample size of individual studies increases in meta-analysis [20]. Therefore, clinical and methodological relevance were the most important issue to consider when deciding whether the results from individual studies could be quantitatively combined in meta-analysis.

Funnel plots were used to assess the association between the point estimates of log odds ratio (a measure of extent of publication bias) and the precision of estimated log odds ratio (inverse of standard errors). The visual assessment of these plots was supported by a formal statistical test using the regression method suggested by Peters et al [21].

## Results

Forty-eight cohort studies provided sufficient data to assess rate of publication by study results. These consisted of 12 inception cohort studies [22-33], four regulatory cohort studies [34-37], 28 abstract cohort studies [38-65], and four manuscript cohort studies [66-69]. Eight inception cohort studies were excluded because they did not provide data on the results of unpublished studies or did not examine the association between publication and study results [12,13,70-75].

### Main characteristics of included cohort studies

The main characteristics of the included cohort studies are summarised by subgroup in four additional files (Additional file 2, 3, 4 and 5). One inception cohort study [32] and four abstract cohort studies [44,49,63,65] were available only in abstract form. Of the 12 cohort studies in the inception subgroup, that assessed the fate of research from its inception, eight did not restrict the field of research, and four were limited to AIDS/HIV [29], health effects of passive smoking [30], complementary medicine [33], or eye diseases [32]. Of the four cohort studies in the regulatory subgroup, two did not specify clinical fields [34,36] and two included studies of anti-depressants [35,37]. All of the cohort studies of conference abstracts were restricted to a specific clinical field, such as emergency medicine, anaesthesiology, perinatal studies, cystic fibrosis, or oncology. Of the four cohorts of journal manuscripts, two examined manuscripts submitted to general medical journals (JAMA, BMJ, Lancet, and Annals of Internal Medicine) [66,69] while two included manuscripts submitted to the American version of the Journal of Bone and Joint Surgery [67,68].

Four of the 12 cohort studies in the inception group, all four studies in the regulatory group, and nine of the 28 cohort studies in the abstract group included only clinical trials. The remaining cohort studies included research of different designs, although separate data for clinical trials was available in some of these studies. Follow-up time ranged from 1 to 12 years in the inception cohort studies, and from 2 to 5 years in the regulatory or abstract cohort studies.

Authors of inception cohort studies used postal questionnaires or telephone interviews of investigators or both to obtain information on results of unpublished studies. The response rate to the survey of investigators ranged from 69% to 92% (see Additional file 2). Information on study results was already available in five regulatory cohort studies and in all abstract cohort studies. Bibliographic databases were usually searched to decide publication status.

Study results were categorised as statistically significant (p < 0.05) or non-significant in 19 studies, and a wide range of different methods were used to categorise study results as, for example, positive versus negative, confirmatory versus inconclusive, striking versus unimportant.

### Pooled estimates of publication bias

**Figure 2:**
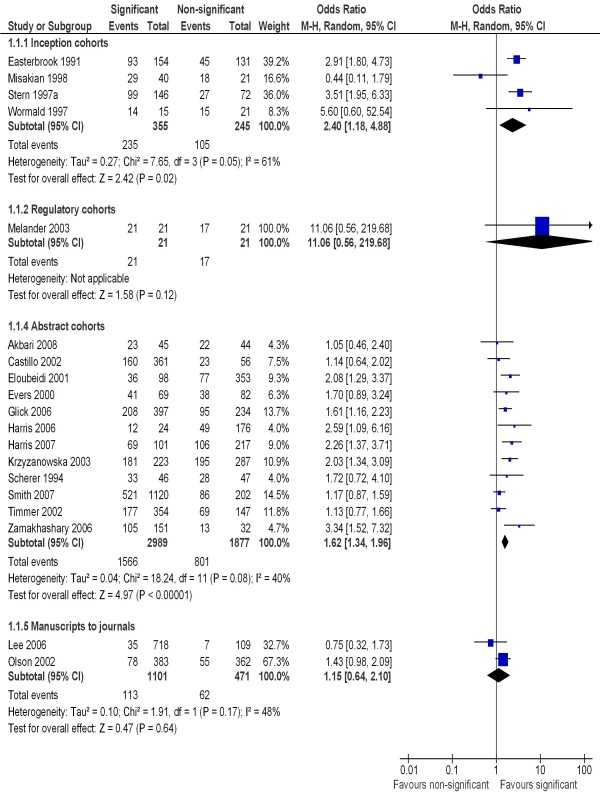
**Rate of publication of statistically significant versus non-significant results - all studies**.

Table 1 summarises the main results of the meta-analyses. The formal publication of statistically significant results (p < 0.05) could be compared with that of non-significant results in four inception cohort studies, one regulatory cohort study, 12 abstract cohort studies and two manuscript cohort studies (Figure 2). The rate of publication of studies in the four inception cohorts ranged from 60% to 93% for significant results and from 20% to 86% for non-significant results. The rate of full publication of meeting abstracts ranged from 37% to 81% for statistically significant results, and from 22% to 70% for non-significant results. Heterogeneity across the four cohort studies from the inception subgroup was statistically significant (I^2 ^= 61%, p = 0.05). There was no statistically significant heterogeneity across studies within the cohort studies of abstracts and cohort studies of manuscripts. The pooled odds ratio for publication bias by statistical significance of results was 2.40 (95% CI: 1.18 to 4.88) for the four inception cohort studies, 1.62 (95% CI: 1.34 to 1.96) for the 12 abstract cohort studies, and 1.15 (95% CI: 0.64 to 2.10) for the two manuscript cohort studies (Figure 2). Only one regulatory cohort study was included in Figure 2, reported an odds ratio of 11.06 (95% CI: 0.56 to 21.9.7) for publication of statistically significant vs. non-significant results.

**Table 1:** Results of meta-analyses of cohort studies of publication bias

Cohort category	No. of cohort studies	Pooled odds ratio (95% CI)	Heterogeneity test: *I*^2 ^(p value)
Statistically significant versus non-significant results			

Inception cohorts	4	2.40 (1.18 to 4.88)	61% (0.05)
Regulatory cohorts	1	11.06 (0.56 to 219.68)	
Abstract cohorts	12	1.62 (1.34 to 1.96)	40% (0.08)
Manuscript cohorts	2	1.15 (0.64 to 2.10)	48% (0.17)

Positive versus non-positive results			

Inception cohorts	12	2.78 (2.10 to 3.69)	37% (0.09)
Regulatory cohorts	4	5.00 (2.01 to 12.45)	64% (0.04)
Abstract cohorts	27	1.70 (1.44 to 2.02)	61% (<0.001)
Manuscript cohorts	4	1.06 (0.80 to 1.39)	22% (0.28)

**Figure 3:**
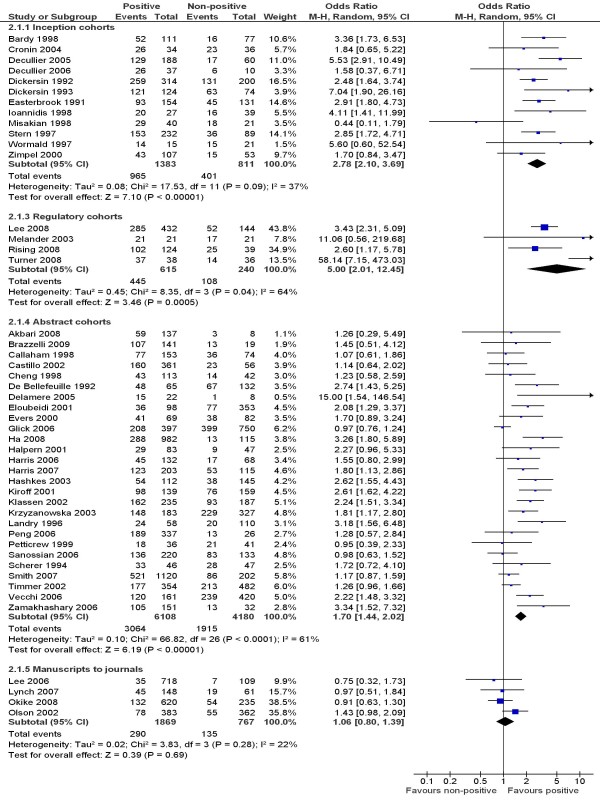
**Rate of publication of positive versus non-positive results - all studies**.

To include data from other cohort studies, a positive result was loosely defined as important, confirmatory or significant, while a 'non-positive' result included negative, non-important, inconclusive or non-significant results. This more inclusive definition of positive results allowed the inclusion of all 12 inception cohort studies, four regulatory cohort studies, 27 abstract cohort studies, and four manuscript cohort studies (Figure 3). There was statistically significant heterogeneity across cohort studies within regulatory (p = 0.04) and abstract subgroups (p < 0.001). Pooled estimates of odds ratios consistently indicated that studies with positive results were more likely to be published than studies with non-positive results, but this was not true after submission for publication (Figure 3).

**Figure 4:**
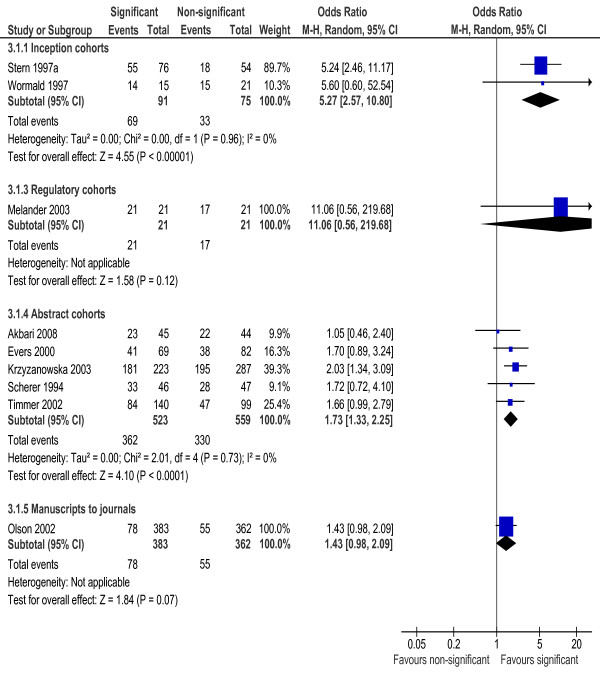
**Rate of publication of statistically significant versus non-significant results - clinical trials only**.

**Figure 5:**
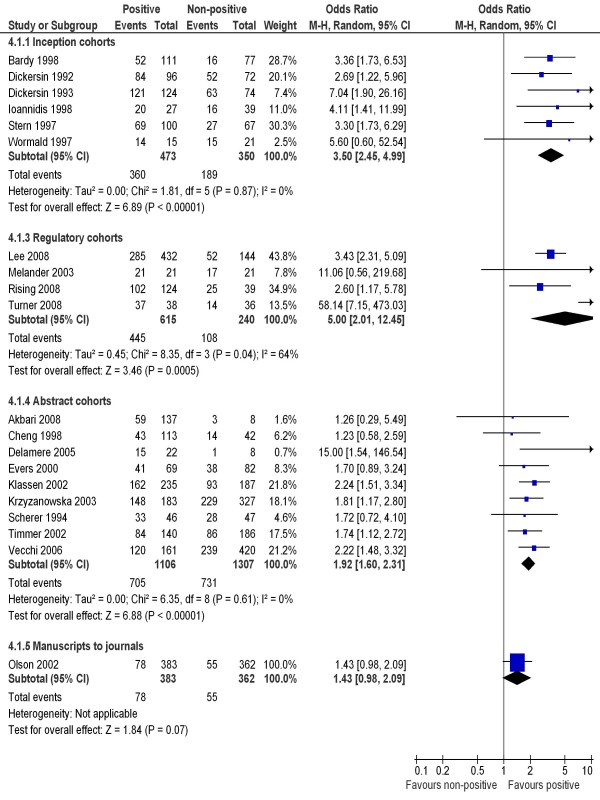
**Rate of publication of positive versus non-positive results - clinical trials only**.

Types of studies included in the cohort studies varied from basic experimental, observational and qualitative research to clinical trials. When the analyses were restricted to clinical trials, the results were similar to that based on all studies, and there was no significant heterogeneity in the extent of publication bias among the included inception cohorts and abstract cohorts of clinical trials (Figure 4 and Figure 5).

**Figure 6:**
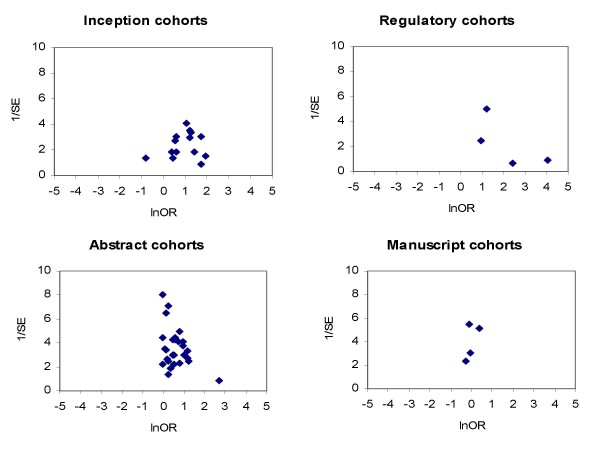
**Funnel plots -- publication of positive and non-positive results**. (Funnel plot asymmetry test: p = 0.178 for inception cohort studies, p = 0.262 for regulatory cohort studies, p = 0.142 for abstract cohort studies, and p = 0.942 for manuscript cohort studies.)

Funnel plots constructed separately for the four subgroups of cohort studies were not statistically significantly asymmetric (Figure 6).

### Factors associated with publication bias

Some cohort studies have examined the impacts of other factors on biased publication of research. The factors investigated include study design, type of study, sample size, funding source, and investigators' characteristics. However, only a few of the included cohort studies reported findings regarding factors associated with publication bias and findings from different studies were often inconsistent.

Easterbrook et al conducted subgroup analyses to examine susceptibility to publication bias amongst various subgroups of studies. They found that observational, laboratory-based experimental studies and non-randomised trials had a greater risk of publication bias than randomised clinical trials. Factors associated with less bias included a concurrent comparison group, a high investigator rating of study importance and a sample size greater than 20 [28].

Dickersin and colleagues investigated the association between the risk of publication bias and type of study (observational, clinical trial), multi or single centre, sample size, funding source and principle investigators' characteristics (such as sex, degree, rank). They found no statistically significant association between any factors examined and publication bias [26]. In a different inception cohort study, Dickersin and Min reported that the odds ratio for publication bias was significantly different between multi-centre versus single centre studies, and between studies with a female principle investigator and studies with a male principle investigator [27]. They did not find an association between publication bias and the use of randomisation or blinding, having a comparison group or a larger sample size [27].

Stern and Simes found that the risk of publication bias tended to be greater for clinical trials (odds ratio 3.13, 95% CI: 1.76 to 5.58) than other studies (for all quantitative studies odds ratio 2.32, 95% CI: 1.47 to 3.66). When analyses were restricted to studies with a sample size ≥100, publication bias was still evident (hazard ratio 2.00, 95% CI: 1.09 to 3.66) [31].

## Discussion

This updated analysis yielded results similar to previous reviews: studies with statistically significant or positive results are more likely to be formally published than those with non-significant or non-positive results [14,16-18]. In 1997, Dickersin combined the results from four inception cohort studies [26-28,31] and found that the pooled adjusted odds ratio for publication bias (publication of studies with significant or important results versus those with unimportant results) was 2.54 (95% CI: 1.44 to 4.47) [16]. A recent systematic review of inception cohort studies of clinical trials found the existence of publication bias and outcome reporting bias, although pooled meta-analysis was not conducted due to perceived differences between studies [18]. A Cochrane methodology review of publication bias by Hopewell et al[17] included five inception cohort studies of trials registered before the main results being known [22,26,27,29,31], in which the pooled odds ratio for publication bias was 3.90 (95% CI: 2.68 to 5.68). In a Cochrane methodology review by Scherer et al, the association between the subsequent full publication and study results was examined in 16 of 79 abstract cohort studies [14], finding that the subsequent full publication of conference abstracts was associated with positive results (pooled OR = 1.28, 95% CI: 1.15 to 1.42).

Our review is the first to enable an explicit comparison of results from cohort studies of publication bias with fundamentally different sampling frames. Biased selection for publication may affect research dissemination over the whole process from before study completion, to presentation of findings at conferences, manuscript submission to journals, and formal publication in journals (Figure 1). It seems that publication bias occurs mainly before the presentation of findings at conferences and before the submission of manuscript to journals (Figure 2 and Figure 3). The subsequent publication of conference abstracts was still biased but the extent of publication bias tended to be smaller as compared to all studies conducted. After the submission of a manuscript for publication, editorial decisions were not clearly associated with study results. However, publication bias may still be an issue for rejected manuscripts, if the possibility of their re-submission to a different journal is associated with the study results. One excluded cohort study (in which data on publication bias was not available) found that psychological research with statistically significant results was more likely to be submitted for publication than research with non-significant results (74% versus 4%) [72].

Since the acceptance of manuscripts for publication by journal editors was not determined by the direction or strength of study results, the existence of publication bias may be largely due to biased selection by investigators of submitted studies. This is supported by data suggesting that a large proportion of submitted papers show significant or positive results (72%) in four cohort studies (Table 2). Since authors inevitably consider the possibility of their manuscripts being rejected before the submission, submitted studies with negative results may be a biased selection of all studies with negative results. In addition, although no conflict of interest was declared in the four cohort studies of submitted manuscripts, this kind of study will always need support or collaboration from journal editors. In prospective studies, editors' decision on manuscript acceptance may be influenced by their awareness of the ongoing study [69]. Therefore, biased selection for publication by journals cannot be completely ruled out. In Olson et al's cohort study of manuscripts submitted to JAMA, there was a tendency that studies with significant results had a higher rate of acceptance than studies with non-significant or unclear results (20.4% vs 15.2%, p = 0.07) [69]. In the cohort study by Okike et al, a subgroup analysis of 156 manuscripts with a high level of evidence (level I or II) found that the acceptance rate was significantly higher for studies with positive or neutral results than studies with negative results (37%, 36% and 5% respectively; p = 0.02) [68].

**Table 2:** Proportion of studies with significant or positive results in studies included in cohort studies of publication bias

	Positive results	
	
Cohort category	%	Total
Inception	63.0%	2,194
Regulatory	71.9%	855
Abstract	59.4%	10,288
Manuscript	70.9%	2,636

Important and convincing evidence on the existence of publication bias comes from the inception cohort studies. Study results were defined differently among the empirical studies assessing publication bias. The most objective method would be to classify quantitative results as statistically significant (p < 0.05) or not. However, this was not always possible or appropriate. When other methods were used to classify study results as important or not, bias may be introduced due to inevitable subjectivity.

Large cohort studies on publication bias were often highly diverse in terms of research questions, designs, and other study characteristics. Many factors (e.g., sample size, design, research question, and investigators' characteristics) may confound the association as they are associated with both study results and the possibility of publication. However, due to insufficient data, it was impossible to exclude the impact of confounding factors on the observed association between study results and formal publication. To improve our understanding of factors associated with publication bias findings from qualitative research on the process of research dissemination may be helpful [76,77].

There was a statistically significant heterogeneity within subgroups of inception, regulatory and abstract cohort studies (Table 1), although restricting analyses to clinical trials reduced the heterogeneity (Figure 4 and 5). The observed heterogeneity may be a result of differences in study designs, research questions, how the cohorts were assembled, definitions of study results, and so on. For example, the significant heterogeneity across inception cohort studies was due to one study by Misakian and Bero (Figure 2 and Figure 3) [30]. After excluding this cohort study, there was no longer significant heterogeneity across inception cohort studies (see Notes to Figure 2 and Figure 3). The cohort study by Misakian and Bero included research on health effects of passive smoking, and the impact of statistical significance of results on publication may be different from studies of other research topics [30].

The four cohorts of trials submitted to regulatory authorities showed a greater extent of publication bias than other subgroups of cohort studies (Figure 3) [34-37]. Only 855 primary studies were included in the regulatory cohort studies, and two of the four regulatory cohort studies focused on trials of antidepressants [35,37]. Therefore, these regulatory cohort studies may be a biased selection of all possible cases.

Studies of publication bias themselves may be as vulnerable as other studies to the selective publication of significant or striking findings [1,78-80]. In this review, the funnel plot asymmetry was non-significant for each of the four research cohorts (Figure 6). However, we identified a large number of reports of full publication of meeting abstracts, and the association between study results and full publication had not been reported in most of these reports. It is often unclear whether this association had not been examined, or was not reported because the association proved to be non-significant. As an example, Zaretsky and Imrie reported no significant difference (p = 0.53) in the rate of subsequent publication of 57 meeting abstracts between statistically significant and non-significant results; but this study could not be included in the analysis due to insufficient data [65].

### Implications

Despite many caveats regarding the available empirical evidence on publication bias, there is little doubt that dissemination of research findings is likely to be a biased process. All funded or approved studies should be prospectively registered and these registers should be publicly accessible. Regulatory authorities should also provide publicly accessible databases of all studies received from pharmaceutical industry. Investigators should be encouraged and supported to present their studies at conferences. A thorough literature search should be conducted in systematic reviews to identify all relevant studies, including searches of registers of clinical trials and available databases of unpublished studies.

## Conclusion

There is consistent empirical evidence that the publication of a study that exhibits statistically significant or 'important' results is more likely to occur than the publication of a study that does not show such results. Indirect evidence indicates that publication bias occurs mainly before the presentation of findings at conferences and the submission of manuscripts to journals.

## Competing interests

The authors declare that they have no competing interests.

## Authors' contributions

FS, YKL, LH, JR, AJS and IH developed the review protocol. SP, JR and FS conducted literature search. SP, FS, LH, YKL, JR and CH extracted and/or checked data from included studies. FS, AJS and IH provided methodological support. FS analysed data and drafted the manuscript. All authors commented on the final manuscript.

## Pre-publication history

The pre-publication history for this paper can be accessed here:

http://www.biomedcentral.com/1471-2288/9/79/prepub

## Supplementary Material

Additional file 1 **Literature search strategies**. Strategies used to search MEDLINE and Cochrane Methodology Register for relevant empirical and methodological studies on publication bias. Click here for file

Additional file 2 **Main characteristics of inception cohort studies**. Shows the main characteristics of studies that followed up cohorts of research from the beginning, including research approved by research ethics committee and those registered by research funding bodies. Click here for file

Additional file 3 **Main characteristics of regulatory cohort studies**. Shows the main characteristics of studies that followed up trials submitted to regulatory authorities. Click here for file

Additional file 4 **Main characteristics of abstract cohort studies**. Shows the main characteristics of studies that followed up abstracts presented at conferences. Click here for file

Additional file 5 **Main characteristics of manuscript cohort studies**. Shows the main characteristics of studies that followed up cohorts of manuscripts submitted to journals for publication. Click here for file
