# Supplementary material for: Extent of publication bias in different categories of research cohorts: a meta-analysis of empirical studies
Source: BMC Med Res Methodol. 2009 Nov 26;9:79. doi: 10.1186/1471-2288-9-79 (PMC2789098; doi:10.1186/1471-2288-9-79)
Supplement: Additional file 1 — Literature search strategies. Strategies used to search MEDLINE and Cochrane Methodology Register for relevant empirical and methodological studies on publication bias. [file 1471-2288-9-79-S1.PDF]

Additional file 1: Literature search strategies for empirical and methodological studies on publication and related biases

MEDLINE Search Strategy

1 *publications/
2 exp publication bias/
3 (bias$ adj3 (publication$ or disseminat$ or language$ or reporting or grey or gray or citation$ or time delay or time lag or national or country or location or conference or abstract or duplicat$ or multiple publication$)).tw,ot.
4 ((reference$ or database$ or index$) adj2 bias$).tw,ot.
5 (file adj drawer$).tw,ot.
6 (time adj2 (completion or publication)).tw,ot.
7 unpublished research.tw,ot.
8 (fail$ adj2 publish$).tw,ot.
9 Or/1-8
10 Limit 9 to yr="1998-2008"

Cochrane Methodology Register Search strategy

1 "Study identification" or
2 "Information retrieval" or
3 "Unpublished data" or
4 "Missing data" or
5 "Updating and cumulative meta-analysis" or
6 "Prospective meta-analysis" or
7 "Small study effects" or
8 "Small trial bias" or
9 "Funding" or
10 "Outcome reporting bias" or
11 "Bias in review" or
12 (bias* NEAR/3 (publication* or disseminat* or language* or reporting or grey or gray or citation* or time delay or time lag or national or country or location or conference or abstract or reference* or index* or database* or duplicat* or multiple publication*)) in Title, Abstract or Keywords
13 from 1998 to 2008 in Cochrane Methodology Register
