# Supplementary material for: Extent of publication bias in different categories of research cohorts: a meta-analysis of empirical studies
Source: BMC Med Res Methodol. 2009 Nov 26;9:79. doi: 10.1186/1471-2288-9-79 (PMC2789098; doi:10.1186/1471-2288-9-79)
Supplement: Additional file 2 — Main characteristics of inception cohort studies. Shows the main characteristics of studies that followed up cohorts of research from the beginning, including research approved by research ethics committee and those registered by research funding bodies. [file 1471-2288-9-79-S2.PDF]

Additional file 2: Main characteristics of included inception cohort studies

Study	Cohort types, Speciality	Study design Follow-up	Verification of publication status and results of unpublished studies	Publication rate	Definition of study results and other notes
Bardy 1998[22]	Clinical trials on medicinal products notified to the National Agency for Medicines in 1987, Finland.<br><br>Mixed speciality	Clinical trials<br><br>Follow-up: 5-6 years	MEDLINE searched for publications.<br><br>Questionnaires sent to trial sponsors for trial results.	Positive 47% (52/111)<br>Inconclusive 33% (11/33)<br>Negative 11% (5/44)	Positive: the drug better (or equivalent to in equivalent trials) than comparators, or the objective of the study supported or confirmed.<br>Inclusive: exploratory studies or non-comparative or the risk-benefit was inconclusive.<br>Publication: published in journals included in Medline.
Cronin & Sheldon 2004 [23]	Studies sponsored by the NHS R&D programme (the North Thames Regional Office) from 07/1995 to 12/1998.<br><br>Mixed speciality	Mixed. Including quantitative (47%) and qualitative (53%) research.<br><br>Follow-up: >2 years	Questionnaires sent to investigators.<br><br>17% failed to respond.	Quantitative or qualitative effect (n=70) published in peer-reviewed journals:<br>Showed an effect 76% (26/34)<br>No effect 64% (23/36)	Methods used in Dickersin[26] were adopted to classify findings.
Decullier et al 2006[25]	Protocols submitted for funding to the G. Lyon regional scientific committee in 1997.<br><br>Mixed speciality	Mixed:<br>Ob=51%<br>CT=25%<br><br>Follow-up: 8 years	Questionnaires sent to investigators, up to 3 times.<br><br>20% failed to respond.	Completed studies:<br>Important results 70% (26/37)<br>Less important 60% (6/10)	Investigators rated the importance of results from 1 to 10. Important results were those >5.

Decullier et al 2005[24]	Biomedical research protocol approved by French RECs in 1994.<br><br>Mixed: biomedical research	Mixed:<br>Ob=13%<br>Exp(CT)=87%.<br><br>Follow-up: 5-7 years	Questionnaires sent to investigators, or from REC databases.<br><br>31% failed to respond.	Confirmatory results 69% (129/188)<br>Invalidating results 19% (3/16)<br>Inconclusive results 32% (14/44)	Confirmatory: results confirming study hypothesis.<br>Invalidating: results invalidating study hypothesis.<br>Inconclusive: not confirming or invalidating.
Dickersin & Min 1993[27]	NIH 1979 funded clinical trials that were completed by 1988.<br><br>Mixed speciality	Clinical trials:<br>CT=100%.<br><br>Follow-up: 9 years	Contacting and telephone interview of investigators.<br><br>26% failed to respond	Sig/important 98% (121/124)<br>Non-significant 85% (63/74)	Significant results: p<0.05 or deemed to be of 'great importance'.<br>Non-significant results: all other results.
Dickersin et al 1992[26]	Studies approved by IRBs at Johns Hopkins Health Institutions up to the end of 1980.<br><br>Mixed speciality	Mixed (Med / Pub):<br>Ob=37% / 85%<br>Exp=17% / 9%<br>CT=46% / 6%.<br><br>Follow-up: >7 years	Telephone interview of investigators.<br><br>30% failed to provide adequate data	Medicine & hospital<br>Sig/important 89% (184/208)<br>Non-significant 69% (93/134)<br><br>Public health<br>Sig/important 71% (75/106)<br>Non-significant 58% (38/66)<br><br>Clinical trials (both centres)<br>Sig/important 87% (84/96)<br>Non-significant 72% (52/72)	Significant results: p<0.05 or results considered to be of great importance.<br>Non-significant results: all other results.<br>Risk of publication bias may be under-estimated by excluding studies due to lack of information.<br>Unpublished data for clinical trials obtained from Hopewell et al 2009.[17]
Easterbrook et al 1991[28]	Studies approved by the Central Oxford REC between 1984-87.<br><br>Mixed speciality	Mixed:<br>Ob=30%<br>Exp=18%<br>CT=52%.<br><br>Follow-up: 3-6 years	Questionnaires sent to investigators, followed by a telephone interview.<br><br>8% failed to respond or provide adequate data	Fully published<br>Significant 60% (93/154)<br>Non-significant trend 35% (12/34)<br>No difference 34% (33/97)<br><br>Published or presented<br>Significant 85% (131/154)<br>Non-significant trend 65% (22/34)<br>No difference 56% (54/97)	Significant results: p<0.05.<br>Non-significant trend: difference with a p value of >/=0.05.<br>Null: no difference.<br><br>Examined factors associated with publication (but not necessarily publication bias)

Ioannidis 1998[29]	RCTs conducted by 2 trialist groups (sponsored by the NIH) from 1986-1996.<br><br>AIDS/HIV	Clinical trial: CT=100%.<br><br>Follow-up: 1-10 years	Information obtained from a database of HIV trials sponsored by NIH. Supplemental data from investigators and staff responsible for the protocols.	Positive results: 74% (20/27)<br>Non-positive: 41% (16/39)	Positive: statistically significant (p<0.05) in favour of an experimental arm.<br>Non-positive: significantly in favour of the control arm or non-significant. The focus of the study was time lag bias. Data obtained from Hopewell et al.[81]
Misakian & Bero 1998[30]	Research on passive smoking funded by 76 organisations between 1981 and 1995.<br><br>Health effects of passive smoking	Mixed:<br>Exp=23%<br>Obs=77%<br><br>Follow-up: median 5 years	Semistructured telephone interview of investigators.<br><br>17% failed to respond	Significant: 85% (28/33)<br>Non-significant: 86% (18/21)<br>Mixed: 14% (1/7)	Statistically significant: p<=0.05. Mixed results: multiple primary outcomes at least one was statistically significant.<br><br>Cox regression analysis was used to estimate hazard ratio.
Stern & Simes 1997[31]	Studies submitted to Royal Prince Alfred Hospital REC between 1979-1988.<br><br>Mixed speciality	Mixed:<br>Ob=22%<br>Exp=22%<br>CT=56% (details available).<br><br>Follow-up: 3-12 years	Questionnaires sent to investigators.<br><br>30% failed to respond.	Quantitative studies<br>Significant results 68% (99/146)<br>Non-significant trend 20% (4/20)<br>No difference 44% (23/52)<br><br>Qualitative studies<br>Striking 70% (19/27)<br>Important/definite 59% (35/59)<br>Negative/unimportant 53% (9/17)<br><br>Clinical trials (n=167)<br>Quantitative trials<br>Significant 72% (55/76)<br>Non-significant trend 20% (3/15)<br>Null 38% (15/39)<br><br>Qualitative trials<br>Striking 50% (3/6)<br>Important/definite 61% (11/18)<br>Negative/unimportant 69% (9/13)	Quantitative studies<br>Significant: p<0.05.<br>Non-significant trend: 0.05</=p<0.10.<br>No difference: p>/=0.10.<br><br>Classification of qualitative studies based on principle investigators' judgement.<br><br>With data on time delayed publication. Qualitative studies similarly vulnerable to publication bias.

Wormald et al 1997[32]	Randomised trials processed through the Pharmacy of Moorfields Eye Hospital since 1963.<br><br>Eye health	RCTs<br><br>Follow-up: >2 years	Retrospective review	Significant 93% (14/15)<br>Non-significant 71% (15/21)	Significant p<0.05; and Non-significant p>=0.05.<br><br>Published as a brief abstract, data from Dwan et al[18]
Zimpel & Windeler 2000 [33]	140 medical theses on complementary medical subjects.<br><br>Complementary medicine	Mixed.<br><br>Follow-up: >5 years	Literature search and contacting investigators.<br><br>Response rate unclear	Positive results: 40% (43/107)<br>Negative results: 28% (15/53)	Full publication in German, information obtained mainly from the abstract.

Notes: REC - Research Ethics Committee. IRB - Institution Research Board. NIH - National Institute of Health. CT – clinical trial. RCT – randomised controlled trial. Ob – observational study. Exp – experimental study.
