# Supplementary material for: Extent of publication bias in different categories of research cohorts: a meta-analysis of empirical studies
Source: BMC Med Res Methodol. 2009 Nov 26;9:79. doi: 10.1186/1471-2288-9-79 (PMC2789098; doi:10.1186/1471-2288-9-79)
Supplement: Additional file 3 — Main characteristics of regulatory cohort studies. Shows the main characteristics of studies that followed up trials submitted to regulatory authorities. [file 1471-2288-9-79-S3.PDF]

Additional file 3: Main characteristics of included regulatory cohort studies

Study	Cohort types, Speciality	Study design Follow-up	Verification of publication status and results of unpublished studies	Publication rate	Definition of study results and other notes
Lee et al 2008[34]	Trials supporting new drugs approved by FDA between 1998 and 2000.<br><br>Mixed speciality	Clinical trials<br><br>Follow-up: >5 years	Searches of PubMed and other databases.<br><br>Trial results obtained from FDA files.	Statistically significant: 66%(285/432)<br>Not statistically sig.: 36%(52/144)	Statistically significance (primary outcome): p<0.05 or if an equivalency trial p>0.05 or a CI excluding the prespecified difference.
Melander et al 2003[35]	RCTs of five SSRIs submitted to the Swedish drug regulatory authority for marketing approval .<br><br>Mental: depression	Clinical trials<br><br>Follow-up: unclear	Literature search for publications.<br><br>Study results based on submitted material.	Stand alone or pooled publications:<br>Significant 100% (21/21)<br>Non-significant 81% (17/21)<br>Stand alone publications:<br>Significant 90% (19/21)<br>Non-significant 29% (6/21)	Also provided data on duplicate, and selective outcome reporting bias.
Rising et al 2008[36]	Efficacy trials supporting new drug applications approved by FDA from 2001 to 2002.<br><br>Mixed speciality	Clinical trials<br><br>Follow-up: >4 years	Search of PubMed and Cochrane Library and contacting sponsors and authors.<br><br>Trial results obtained from FDA files.	<u>Primary outcomes</u><br>Favourable: 82% (102/124)<br>Not favourable: 66% (19/29)<br>Unknown: 60% (6/10)<br><br><u>Conclusion</u><br>Favourable: 79% (90/114)<br>Not favourable: 64% (7/11)<br>Unknown: 57% (4/7)	Favourable: statistically significantly in favour of the new drug or equivalence was found.<br><br>Note: pooled publication not reported separately was considered "not published".
Turner et al 2008[37]	Trials of 12 antidepressants, submitted to FDA between 1987 and 2004.<br><br>Mental: depression	Phase 2 and 3 clinical trials.<br><br>Follow-up: >2 years	Literature search and contacting sponsors for publications.<br><br>Trial results obtained from FDA files.	Positive results: 97% (37/38)<br>Questionable: 50% (6/12)<br>Negative results: 33% (8/24)	Trial results were classified according to the FDA's regulatory decisions.

Nates: FDA **-** US Food and Durg Administration. SSRI **-** Selective Serotonin Reuptake Inhibitor. RCT **-** Randomized Controlled Trial
