# Supplementary material for: Extent of publication bias in different categories of research cohorts: a meta-analysis of empirical studies
Source: BMC Med Res Methodol. 2009 Nov 26;9:79. doi: 10.1186/1471-2288-9-79 (PMC2789098; doi:10.1186/1471-2288-9-79)
Supplement: Additional file 4 — Main characteristics of abstract cohort studies. Shows the main characteristics of studies that followed up abstracts presented at conferences. [file 1471-2288-9-79-S4.PDF]

Additional file 4: Main characteristics of included abstract cohort studies

Study	Cohort types, Speciality	Study design Follow-up	Verification of publication status and results of unpublished studies	Publication rate	Definition of study results and other notes
Akbari-Kamrani et al 2008[38]	Presented meeting abstracts<br><br>Laser medicine and surgery	Clinical trials<br><br>Follow-up: >3 years	Literature search for full publications.	Significant (p<0.05): 51% (23/45)<br>Non-significant: 50% (22/44)<br><br>Positive: 43% (59/137)<br>Not-positive: 38% (3/8)	Positive: if stated the intervention has some beneficial effect. Negative: against the intervention. Equivocal: no statement about the effectiveness or the comparison groups considered the same.
Brazzelli et al 2009[39]	Presented meeting abstracts<br><br>Stroke	Studies of diagnostic tests<br><br>Follow-up: >2 years	Literature search and contacting authors for full publications.	Clinical utility:<br>Accurate: 76% (107/141)<br>Possibly or non-informative: 68% (13/19)<br><br>Sensitivity (median 0.91)<br>Above median: 77% (38/49)<br>Below median: 74% (34/46)<br>Not given: 75% (49/65)<br><br>Specificity (median 0.91)<br>Above median: 71 (30/42)<br>Below median: 73% (27/37)<br>Not given: 79% (64/81)	Accurate: diagnostic accuracy of the test was high enough to recommend its use in clinical practice. Possible useful: having a good sensitivity but not necessarily a good specificity (and vice versa). Non-informative: the accuracy of the test was not good enough to recommend its use in clinical practice or equivalent to or not better than that of an existing alternative test.
Callaham et al 1998[40]	Submitted meeting abstracts<br><br>Emergency medicine	Mixed:<br>CT=26%.<br><br>Follow-up: 5 years	Literature search and contacting authors for full publications.	Positive : 50% (77/153)<br>Not positive: 49% (36/74)	Positive results: beneficial results or p<0.05.<br><br>Publication rates obtained from Scherer et al.[14]
Castillo et al 2002[41]	Presented meeting abstracts.<br><br>Anaesthesiology	Mixed:<br>Ob=69%<br>RCT=31%.<br><br>Follow-up: 4-5 years	Literature search for full publications.	Significant results 44% (160/361)<br>Non-significant 41% (23/56)	Significant: p<0.05.

Cheng et al 1998[42]	Abstracts from 3 international conferences over a 30 yr period.<br><br>Cystic fibrosis	Clinical trials: CT=100%.<br><br>Follow-up: not reported	Literature search for full publications	Positive results 38% (43/113)<br>Negative 33% (14/42)	Positive: authors concluded the test treatment was superior to control or equally effective (in equivalence trials).<br><br>Publication rates from Scherer et al. log-rank tests showed no significant difference in time to publication between 'positive' or 'negative' results ($p=0.54$).
De Bellefeuille et al 1992[43]	Submitted meeting abstracts.<br><br>Clinical oncology	Mixed: CT=48%.<br><br>Follow-up: 5 years	Literature search and contacting authors for full publications.	Positive 74% (48/65)<br>Negative 32% (10/31)<br>Neutral/descriptive 56% (57/101)	Positive results: $p<0.05$ or beneficial to interventions.
Delamere & Williams 2005[44]	Conference abstracts.<br><br>Dermatology	Clinical trials: CT=100%.<br><br>Follow-up: 3-5 years	Literature search for full publications.	Positive 68% (15/22)<br>Negative 0% (0/2)<br>Neutral 17% (1/6)	Unclear definition of positive or negative results. Only abstract available.
Eloubeidi et al 2001[45]	Submitted meeting abstracts.<br><br>Gastro-intestinal endoscopy	Mixed: RCT=9%.<br><br>Follow-up: 4 years	Literature search for full publications.	Significant 37% (36/98)<br>Non-significant 22% (77/353)	Positive: statistically significant $p<0.05$.<br><br>Multivariate adjusted OR: 0.97 (0.58-1.60); HR=1.92 (1.28-2.87). Also with data on presentation acceptance.
Evers 2000[46]	Presented meeting abstracts.<br><br>Reproductive	RCTs=100%.<br><br>Follow-up: 4-8 years	Literature search for full publications	Significant results 59% (41/69)<br>Non-significant 46% (38/82)	Significant: $p<0.05$.
Glick et al 2006[47]	Presented meeting abstracts.<br><br>Organ trans-plantation	Mixed: Ob=81% Other=13% RCT=6%.<br><br>Follow-up 4-5 years	Literature search for full publications	Significant results 52% (208/397)<br>Not specified 59% (304/516)<br>Non-significant 41% (95/234)	Significant $p<0.05$.<br><br>Statistical significance was excluded from multivariate analysis due to >5% of data missing.
Ha et al 2008[48]	Presented meeting abstracts.<br><br>Radiology	Mixed<br><br>Follow-up: >4 years	Literature search for full publications	Positive: 29% (288/982)<br>Negative: 11% (13/115)	Positive outcomes: beneficial or statistically significant results. Negative: non-positive results.

Halpern 2001[49]	Presented meeting abstracts.<br><br>Anaesthesiology	Mixed<br><br>Follow-up: >5 years	Literature search for full publications	Positive results: 35% (29/83)<br>Not positive: 19% (9/47)	Positive results: significant results. Lack of details. Unpublished data reported in Scherer et al.[14]
Harris et al 2006[51]	Presented meeting abstracts.<br><br>Orthopaedic	Mixed:<br>Ob=72%<br>Other=26%<br>RCT=2%.<br><br>Follow-up: 5 years	Literature search and contacting authors for full publications	Positive results 34% (45/132)<br>Negative 50% (5/10)<br>Neutral 21% (12/58)<br><br>Significant (p<0.05) 50% (12/24)<br>Non-significant 28% (50/176)	Positive results: beneficial regardless of p values.<br>Negative results: against the intervention.<br>Neutral results: no opinion.<br><br>After adjusting for study setting (clinical or laboratory), neither statistical significance (p=0.2) nor direction of outcome (p=0.3) were significantly associated with publication.
Harris et al 2007[50]	Presented meeting abstracts.<br><br>Orthopaedic	Mixed:<br>Ob=82%<br>Other=12%<br>RCT=6%.<br><br>Follow-up: 5 years	Literature search and contacting authors for full publications	Positive results 61% (123/203)<br>Negative 53% (18/34)<br>Neutral 43% (35/81)<br><br>Significant (p<0.05) 68% (69/101)<br>Non-significant 49% (107/217)	Positive results: beneficial regardless of p values.<br>Negative results: against the intervention.<br>Neutral results: no opinion.
Hashkes & Uziel 2003[52]	Presented meeting abstracts.<br><br>Paediatric rheumatology	Mixed:<br>Basic=5%<br>Ob=69%<br>Analytic/ CT=26%.<br><br>Follow-up: 3 years	Literature search and contacting authors for full publications	Positive 48% (54/112)<br>Negative 14% (2/14)<br>Neutral 27% (36/131)	Definition of positive, negative or neutral results not provided.
Kiroff 2001[53]	Presented meeting abstracts.<br><br>Surgery	Mixed:<br>RCT=4%<br>CT=31%.<br>(Subgroup - clinical trials).<br><br>Follow-up: 3-5 years	Contacted authors for full publications.	All studies:<br>Positive 71% (98/139)<br>Negative/inclusive 48% (76/159)<br><br>Clinical trials:<br>Positive 92% (11/12)<br>Negative/inclusive 50% (4/8)	Significance or importance of the results based on information from authors, but lack of details.<br><br>A single investigator assessed meeting abstracts.

Klassen et al 2002[54]	Presented meeting abstracts.<br><br>Paediatric	RCT=100%.<br><br>Follow-up: 5-8 years	Literature search for full publications	Favouring treatment 69% (162/235)<br>Non-favourable 50% (93/187)	Favouring treatment: overall conclusions favoured the intervention.<br><br>With data on time to publication, and abstract bias.
Krzyzanowska et al 2003[55]	Presented meeting abstracts.<br><br>Oncology	Large clinical trials (n>200):<br>CT=100%.<br><br>Follow-up: <5 years	Literature search and contacting authors for full publications	Significant results 81% (181/223)<br>Non-significant 68% (195/287)<br><br>Positive results 81% (148/183)<br>Negative 70% (229/327)	Significant results: p</=0.05.<br>Positive results: p</=0.05 in favour of the experimental treatment.<br><br>With data on time to publication.
Landry 1996[56]	Presented meeting abstracts.<br><br>Burn research	Mixed:<br>CT=27%.<br><br>Follow-up: 4 years	Literature search for full publications	Positive results 41% (24/58)<br>Non-positive 18% (20/110)	Positive: p<0.05 or stated to be positive.<br><br>Data not clearly presented. Publication rate from Scherer et al.
Peng et al 2006[57]	Presented meeting abstracts.<br><br>Otolaryngology: head and neck surgery	Mixed<br><br>Follow-up: >5 years	Literature search for full publications	Positive results 56% (189/337)<br>Negative results 50% (13/26)	Unclear definition of positive or negative results.
Petticrew et al 1999[58]	Presented meeting abstracts.<br><br>Social medicine	Mixed:<br>CT=5%.<br><br>Follow-up: 2 years	Literature search and contacting authors for full publications	Positive results 50% (22/36)<br>Uncertain 56% (19/34)<br>Negative 57% (4/7)	Classification of results based on subjective assessment of the study results and the authors' conclusions.
Sanossian et al 2006[59]	Presented meeting abstracts.<br><br>Stroke	Mixed:<br>CT=2%.<br><br>Follow-up: 5 years	Literature search and contacting authors for full publications	Positive results 62% (136/220)<br>Non-positive 62% (83/133)	Positive: beneficial or supported hypothesis or objective, and either p<0.05 or no statistical test reported.<br><br>Adjusted publication rate: 64% for positive and 59% for non-positive results. Clinical trials 100% published.
Scherer et al 1994[60]	Presented meeting abstracts.<br><br>Ophthalmology	Clinical trials:<br>CT=100%.<br><br>Follow-up: 3 years	Literature search and contacting authors for full publications	Significant 72% (33/46)<br>Non-significant 59% (28/47)	Statistically significant p<0.05.

Smith et al 2007[61]	Presented meeting abstracts<br><br>Urology	Mixed clinical research<br><br>Follow-up: >2 years	Literature search for full publications	Significant: 47% (521/1120)<br>Non-significant: 43% (86/202)	Positive results were those showing statistically significant results (p<0.05) regardless of the direction.
Timmer et al 2002[62]	A random sample of abstracts submitted to a conference<br><br>Gastroenterology	Controlled clinical trials (39%), other clinical research (40%) and basic studies (21%).<br><br>Follow-up: 3-6 years	Literature search for full publications	<u>All abstracts</u><br>Significant (p<0.05): 50% (177/354)<br>Non-significant: 47% (69/147)<br>Equivocal: 43% (144/335)<br><br><u>Controlled clinical trials</u><br>Significant (p<0.05): 60% (84/140)<br>Non-significant: 48% (47/99)<br>Equivocal: 45% (39/87)	Significant results: p<0.05.<br>Equivocal results: no statements concerning the statistical significance of the main or the majority of outcomes.
Vecchi et al 2006[63]	Presented meeting abstracts.<br><br>Drug addiction	Clinical trials: CT=100%.<br><br>Follow-up: >5 years	Literature search for full publications	Significant (p<0.05) 74% (182/245)<br>Non-significant 65% (85/131)	Abstract only. Unclear about no quantitative results and negative results. Also investigated time to publication.
Zamakhashary et al 2006[64]	Presented meeting abstracts.<br><br>Paediatric	Mixed:<br>Basic=25%<br>Ob=36%<br>RCT=1%.<br><br>Follow-up: <2 years	Literature search for full publications	Significant (p<0.05) 70% (105/151)<br>Non-significant 41% (13/32)	Significant results: p<0.05.
Zaretsky et al 2002[65]	Presented meeting abstracts.<br><br>Haematology	Phase III trials (n=57).<br><br>Follow-up: 7 years	Literature search for full publications	The rates of publication of positive and negative results not significantly different (p=0.53)	Only a short abstract available.

Notes: CT – clinical trial. RCT – randomised controlled trial. Ob – observational study. Exp – experimental study.
