# Supplementary material for: Extent of publication bias in different categories of research cohorts: a meta-analysis of empirical studies
Source: BMC Med Res Methodol. 2009 Nov 26;9:79. doi: 10.1186/1471-2288-9-79 (PMC2789098; doi:10.1186/1471-2288-9-79)
Supplement: Additional file 5 — Main characteristics of manuscript cohort studies. Shows the main characteristics of studies that followed up cohorts of manuscripts submitted to journals for publication. [file 1471-2288-9-79-S5.PDF]

Additional file 5: Main characteristics of included manuscript cohort studies

Study	Methods	Main findings
Olson et al 2002[69]	Cohort study of 745 manuscripts of controlled trials submitted to JAMA from 02/1996-08/1999.<br><br>Outcome classification:<br>1. Statistically significant ($p<0.05$) for the primary outcome<br>2. Statistically non-significant<br>3. Unclear	Proportion of studies with different results:<br>- 51.4% (n=383) with significant results<br>- 45.7% (n=341) with non-significant results<br>- 2.8% (n=21) with unclear results.<br><br>Acceptance rate:<br>- 20.4% (78/383) for significant results<br>- 15.0% (51/341) for non-significant results<br>- 19.0% (4/21) for unclear results.<br><br>Logistic regression analysis: significant vs non-significant results OR=1.30 (95% CI 0.87 to 1.96)
Lee et al 2006[66]	Cohort study of 1107 manuscripts of original research (including qualitative research, excluding single case reports) submitted to BMJ, Lancet and Annals of Internal Medicine between 01-03/2003 and between 11/2003-02/2004.<br><br>Outcome classification:<br>1. Statistically significant ($p<0.05$) for the primary outcome<br>2. Non-significant	Proportion of different statistical results:<br>- 86.8% (n=718) with significant results<br>- 13.2% (n=109) with non-significant results<br><br>Acceptance rate:<br>- 4.9% (35/718) for significant results<br>- 6.4% (7/109) for non-significant results.<br><br>Multivariate analysis: OR=0.83 (95% CI 0.34 to 1.96).
Lynch et al 2007[67]	Cohort study of 209 manuscripts of original research on hip or knee arthroplasty submitted to The Journal of Bone and Joint Surgery (American Volume) between 01/2004-06/2005.<br><br>Outcome classification:<br>1. Positive or favourable or significant<br>2. Negative or nonsupportive or no difference<br>3. Not analyzable - unclear	Proportion of studies with different results:<br>- 70.8% (n=148) with positive results<br>- 23.4% (n=49) with negative results<br>- 5.7% (n=12) with unclear results.<br><br>Acceptance rate:<br>- 30..4% (45/148) for positive results<br>- 36.7% (18/49) for negative results<br>- 8.3% (1/12) for unclear results.<br><br>Difference in publication rate between positive and negative outcomes was not statistically significant ($p=0.41$).
Okike et al 2008 [68]	Cohort study of 855 manuscripts as scientific articles submitted to the Journal of Bone and Joint Surgery (American Version) between 01/2004-06/2005.<br><br>Outcome classification:<br>1. Positive – favoured experimental item<br>2. Negative – favoured existing standard of care over the experimental item<br>3. Neutral – no difference<br>4. Not applicable	Proportion of studies with different results:<br>- 72.5% (n=620) with positive results<br>- 12.3% (n=105) with negative results<br>- 15.2% (n=130) with neutral results.<br><br>Acceptance rate:<br>- 21.3% (132/620) for positive results<br>- 21.0% (22/105) for negative findings<br>- 24.6% (32/130) for neutral results<br><br>Multivariate analysis: positive vs nonpositive OR=0.92 (95% CI 0.62 to 1.35).
